# Evaluation of mortality prediction using SOFA and APACHE IV tools in trauma and non-trauma patients admitted to the ICU

**DOI:** 10.1186/s40001-022-00822-9

**Published:** 2022-09-30

**Authors:** Mehran Karami Niaz, Nazanin Fard Moghadam, Abbas Aghaei, Saeed Mardokhi, Somayeh Sobhani

**Affiliations:** 1 Student Research Committee, Kurdistan University of Medical Sciences, Sanandaj, Iran; 2 Social Determinants of Health Research Center, Research Institute for Health Development, Kurdistan University of Medical Sciences, Sanandaj, Iran; 3 Department of Epidemiology and Biostatistics, Faculty of Medicine, Kurdistan University of Medical Sciences, Sanandaj, Iran; 4 Kowsar Medical, Educational and Treatment Center, Kurdistan University of Medical Sciences, Sanandaj, Iran

**Keywords:** Mortality, Prediction, ICU, APACHE IV, SOFA

## Abstract

**Background:**

Various tools have previously been introduced to predict the recuperation and mortality of patients in intensive care units and to classify them, which have particular advantages and disadvantages compared to each other. The present study compared the prediction power of mortality of trauma and non-trauma patients admitted to the ICU by SOFA and APACHE IV tools.

**Methods:**

In this retrospective cohort study, patients admitted to the ICU of Kowsar Hospital in Sanandaj from the beginning of April 2020 to the end of December 2020 were assessed. Data were collected in the form of a questionnaire based on APACHE IV and SOFA criteria as well as the demographic information questionnaire. All collected data related to the first 24 h of patients' hospitalization was analyzed in SPSS V16 software using Chi-square, Mann–Whitney, Cox regression and Pearson correlation coefficient.

**Results:**

This study was performed on 404 patients admitted to the ICU, Out of which 273 people (67.6%) were male, 208 (51.5%) trauma patients and 196 (48.5%) non-trauma ones. Patients’ mean age was 54.76 ± 20.77 years and their average length of stay in the hospital was 10.05 ± 8.49 days. In general, the AUC obtained by APACHE IV tool (0.902) was slightly better than that of SOFA tool (0.895). However, in a specific study of traumatic and non-traumatic patients, it was found that APACHE IV and SOFA tools had better performance in predicting death innon-trauma and trauma patients based on the accuracy, AUC and sensitivity, respectively.

**Conclusions:**

Based on the results of this study, the difference between APACHE IV and SOFA tools in predicting death of patients admitted to the ICU was very small but the function of APACHE IV was better in predicting mortality of non-traumatic patients, while the function of SOFA was better in predicting the death of traumatic cases. This represents the applicability of these two tools in different patient subgroups.

## Background

Critically patients with severe condition are always at risk of health-threatening risks. Therefore, they need more specialized and vigilant nursing care. The most appropriate department for the care of these patients is the intensive care unit [1]. Patients admitted to this ward need advanced and high quality care due to the high variety of acute and critical illnesses [2]. It is only in these circumstances that these patients benefit from hospitalization in the intensive care unit rather than general wards [3]. New and advanced technology allows us to treat many diseases in the ICU and lead to patients’ longer and further survival [4]. About 13% of hospital costs and 4.2% of health care costs are related to care provided in intensive care units and it should be noted that long-term hospitalization in the ICU is one of the important factors of the increase in costs [5]. Given that the classification of disease severity in terms of service delivery prevents many adverse events and increases the likelihood of patient survival, a valid indicator is necessary to assess the status of patients admitted to the intensive care unit [6]. It should also be noted that the evaluation indicator is also important from the management perspective, because it can be used to make accurate decisions about patients' prioritization in terms of receiving special services, applying manpower and occupying hospital beds. Despite the introduction of effective evaluation indicators in recent years, in intensive care units of many hospitals and training centers, only GCS^1^tools and vital signs are still used to evaluate patients [7].

Today, several tools have been proposed, each of which has its own advantages and disadvantages. They have also been updated and compared through studies. Among these, the tools frequently used are the SAPS^2^, APACHE^3^, MPM^4^, and SOFA^5^ systems [8, 9]. In 1981, the first classification system for disease severity was introduced by University of Washington called APACHE. Subsequent newer versions, such as APACHE II^6^ in 1985 and SAPS in 1993 were invented and have been widely used to date [10, 11]. APACHE IV^7^ was later introduced in 2006 [12], which has been used to estimate the probability of short-term mortality as well as to predict the length of stay in the intensive care unit using clinical data during the first 24 h of hospitalization [13]. With the introduction of APACHE IV in 2006, older models have been suggested to not be used for long periods of time because of the decrease in accuracy of their results [7]. Due to the obvious results, APACHE IV is a statistically appropriate criterion in patients' mortality prediction [14]. This criterion evaluates critically ill patients taking into account various parameters, such as physiological variables, vital signs, urinary outputs, nerve scores, age and other conditions which may have a significant effect on the results [6]. The SOFA scoring system was introduced in 1996 by Vincent et al. to assess organ failure. This system examines six organs, such as pulmonary, blood, cardiovascular, hepatic, central nervous and renal ones [9]. This tool (SOFA) systematically and continuously evaluates the condition of organs in the body during the patient stay in the intensive care unit [15]. In the SOFA scoring system, scores from 0 to 4 are awarded to each of the 6 examined organs based on the level of their dysfunction. Since its introduction, SOFA is being routinely used in the intensive care units to predict the morbidity and mortality of the patients [16].

Considering that the disease severity classification in terms of receiving services can prevent many adverse events and increase patients’ survival, a valid indicator seems necessary to evaluate patients admitted to the intensive care unit [17]. Due to the importance of this issue, the limited equipment, and insufficiency of financial and personnel resources of hospitals, in this study, the observed mortality rate was compared with the rate predicted by APACHE IV and SOFA tools.

## Methods

In this retrospective cohort study, patients admitted to the ICU of Kowsar Hospital in Sanandaj from the beginning of April 2020 to the end of December 2020 were examined. Exclusion criteria included age less than 18 years or over 95 years, duration of ICU hospitalization less than 24 h, pregnancy, case defects based on the criteria required to complete the APACHE IV and SOFA questionnaires and infection with COVID-19. Data were collected as a questionnaire based on APACHE IV and SOFA criteria and the demographic information questionnaire.

The first part of the questionnaire included demographic information, such as sex (female/male), patient’s status in terms of trauma or non-trauma and the length of stay in the hospital (days). The second part of the questionnaire included the APACHE IV questionnaire consisting several sub-sets (sections), in the first part of which the average temperature (Celsius), mean arterial blood pressure (mm Hg), heart rate per minute, number of breaths per minute and respiratory dependence status (connected to the ventilator or not) as well as the amount of sodium (mEq/L), glucose (mg/dL), creatinine (mg/dL), urea (mEq/L), hematocrit (%) and white blood cell count were recorded. In addition, the 24-h urine volume (mL/24 h), blood albumin (g/L), bilirubin (mg/dL), FiO2 (%), PH, PaO2 (mmHg) and PCO2 (mmHg) were recorded in the APACHE IV table. The second part of the table was related to the patient's level of consciousness based on GCS [3–15] in which the GCS criteria, i.e., visual, verbal and motor responses of the patient, were reviewed and recorded. The third part of the questionnaire was related to the SOFA questionnaire which included the parameters of FiO2, PaO2, GCS, Vasopressors (Dopamine, Dobutamine, Epinephrine and Norepinephrine), creatinine, 24-h urine volume, respiratory dependence status, platelets and mean arterial pressure. The parameters entered in the questionnaires were related to the first 24 h. In the third part, the patient's age (years) and in the fourth part, his/her chronic health conditions including CRF,^8^ AIDS,^9^ immunodeficiency, liver failure, lymphoma, metastatic cancer, leukemia and multiple myeloma were examined and recorded. In the fifth part, the patient’s ICU admission information including the need for an emergency surgery and the referral procedure were registered. In the sixth part, information about the initial diagnosis at the time of hospitalization time was recorded.

Data was analyzed by SPSS V16 software using Chi-square, Mann–Whitney, Cox regression and Pearson correlation coefficients.

## Results

This study was performed on 404 patients admitted to the ICU, of whom 273 people (67.6%) were male, 131 (32.4%) female, 208 (51.5%) trauma patients and 196 (48.5%) non-trauma ones. The mean age of the patients was 54.76 ± 20.77 years and their average length of stay in the hospital was 10.05 ± 8.49 days.

Information on demographic variables and scores of APACHE IV and SOFA indicators in terms of death and discharge (transfer to normal wards from the ICU) by differentiating patients to traumatic or non-traumatic have been given in Table 1.

**Table 1:** Comparison of the demographic variables and scores of the two indicators of APACHE IV and SOFA in terms of death and discharge in the ICU by trauma

Variable		Trauma		*P* Value	Non-trauma		*P* Value	Total		^a^*P* Value
		Discharge	Death		Discharge	Death		Discharge	Death	
Sex number (%)	Male	128 (81.0)	(19.0) 30	0.053	(76.5) 88	27 (23.5)	0.204	(79.1) 216	57 (20.9	0.772
	Female	(68.0) 34	(32.0) 16		(84.0) 68	13 (16.0)		102 (77.9)	29 (22.1)	
Age (year) mean (IQR)		40.5 (28.8–65)	64.0 (44.5–72.5)	> 0.001	61.0 (44.0–73.0)	71.0 (61–82.5)	> 0.001	52.5 (34.0–69.0)	67.5 (54.5–76.2)	> 0.001
Length of stay in the ICU (day), mean (IQR)		7.5 (5.0–13.0)	9.5 (5.0–17.0)	0.180	6.0 (4.0–10.0)	9.5 (4.2–18.0)	0.032	7.0 (5.0–11.0)	9.5 (5.0–17.2)	0.012
Glasgow index, mean (IQR)		13 (11.0–14.0)	6.0 (3.8–9.0)	> 0.001	14.0 (12.2–14.4)	8.0 (4.0–10.0)	> 0.001	13.0 (11.0–14.0)	7.0 (4.0–10.0)	> 0.001
APACHE IV score, mean (IQR)		43.0 (30.0–56.0)	80.5 (66–95.8)	> 0.001	46.0 (34.2–60.0)	85.5 (74.2–103.)	> 0.001	44.0 (31.75–58.0)	81.0 (62–98.2)	> 0.001
APACHE IV mortality prediction, mean (IQR)		6.7 (3.6–14.1)	44.2 (31.8–62.3)	> 0.001	7.7 (3.7–13.9)	50.0 (29.5–66.7)	> 0.001	6.95 (3.68–14/03)	45.5 (30.8–64-9)	> 0.001
SOFA score, mean (IQR)		3.0 (2.0–4.0)	9.0 (7.0–11.0)	> 0.001	3.0 (2.0–4.0)	7.0 (5.0–10)	> 0.001	3.0 (2.0–4.0)	8.5 (6.7–11.0)	> 0.001
SOFA mortality prediction percentage, mean (IQR)		5.0 (5.0–10-0)	20.0 (20.0–50.0)	> 0.001	5.0 (5.0–10-0)	20.0 (10.0–50.0)	> 0.001	5.0 (5.0–10-0)	20.0 (17.5–50.0)	> 0.001

^a^Qualitative variables were compared based on the chi-square test and quantitative variables based on the Mann–Whitney test

There was no significant difference between the outcomes of death and discharge among men and women (P=0.772). In addition, the relationship between the number of hospitalization days and the outcome of death and discharge in trauma patients was not significant (P=0.180). According to Table 1, the relationship between APACHE IV and SOFA scores, GCS scores and the outcomes was significant in all patients (P < 0.001).

**Fig. 1:**
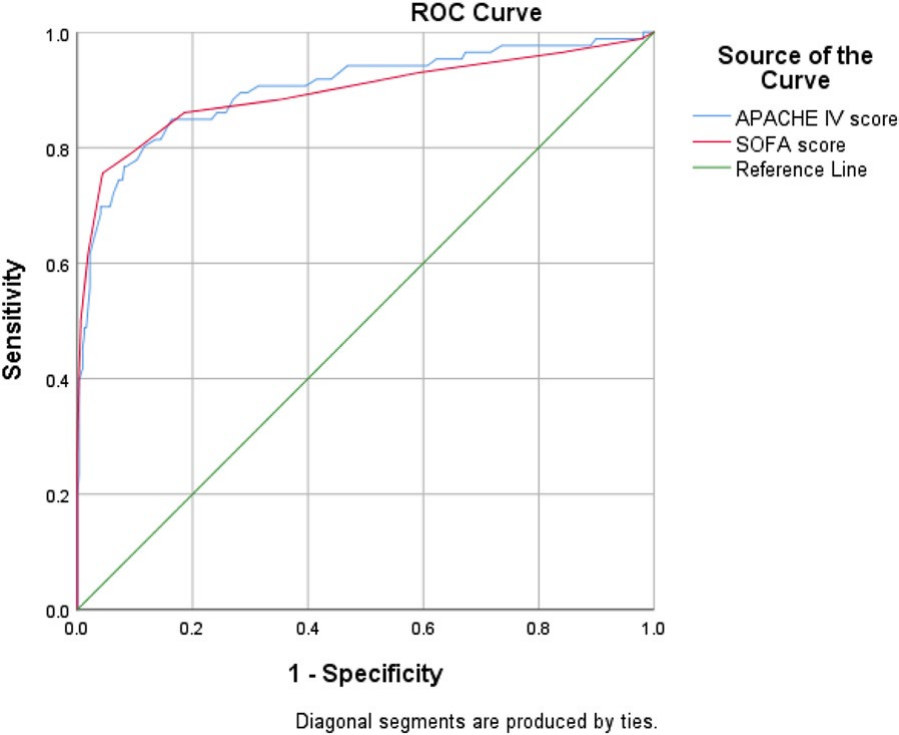
Areas under the APACHE IV and SOFA rock curves based on mortality observed in the ICU

**Fig. 2:**
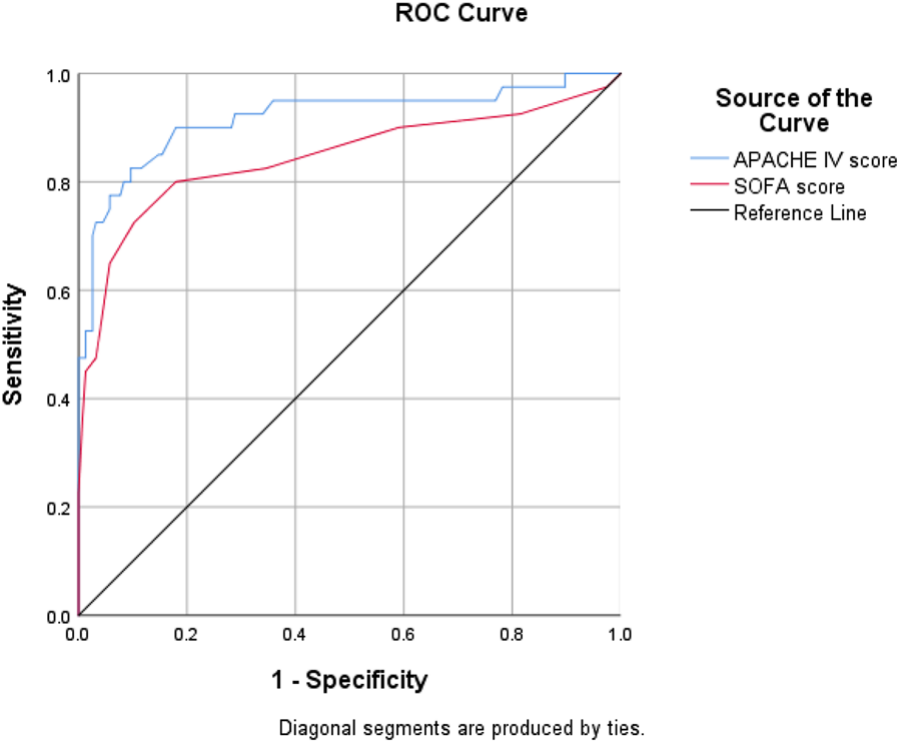
Areas under the APACHE IV and SOFA rock curves based on mortality observed in non-trauma patients in the ICU

**Fig. 3:**
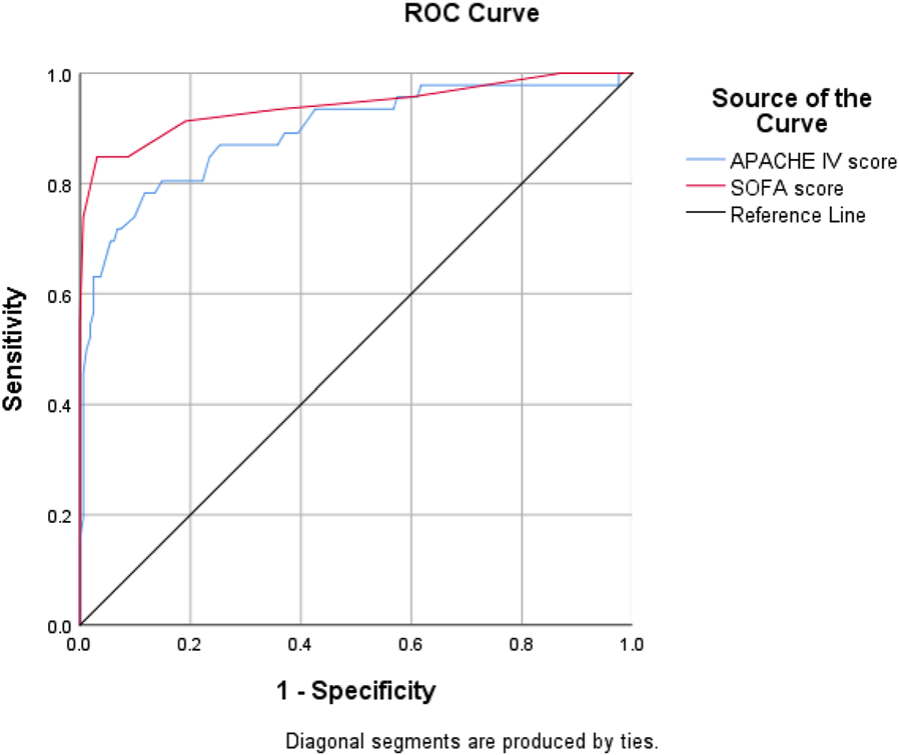
Areas under the APACHE IV and SOFA rock curves based on mortality observed in trauma patients in the ICU

In Fig. 1, the area under the rock curve was examined for the two studied tools based on the mortality rate observed in all patients. As can be seen in Table 2, the AUC^10^value for APACHE IV tool was 0.902 and for SOFA tool was 0.895. To differentiate patients’ conditions, in Figs. 2 and 3, the areas under the rock curves were presented for APACHE IV and SOFA tools. The order was based on the death rate observed in non-trauma patients (Fig. 2) and trauma ones (Fig. 3).

**Table 2:** Area under the rock curve, sensitivity and specificity of APACHE IV and SOFA tools in predicting the outcome of mortality in the ICU patients by differentiating traumatic and non-traumatic patients

Tool	AUC	Lower bound of AUC	Upper bound of AUC	Cutoff point	Accuracy (%)	Sensitivity (%)	Specificity (%)
APACHE IV	0.902	0.858	0.946	74	90.35	69.77	95.91
SOFA	0.895	0.846	0.944	7	91.34	75.58	95.60
APACHE IV							
Trauma	0.893	0.832	0.954	75	89.90	63.4	97.53
Non-trauma	0.918	0.857	0.978	78	91.84	72.5	96.79
SOFA							
Trauma	0.940	0.890	0.989	7	94.23	84.78	96.91
Non-trauma	0.845	0.759	0.931	7	88.27	65.0	94.23

According to the values ​​obtained in Table 2 and according to Figs. 2 and 3, the AUC based on mortality observed in non-traumatic patients was 0.918 for APACHE IV tool and 0.845 for SOFA tool. In addition, AUC based on deaths observed in trauma patients was 0.893 for APACHE IV tool and 0.940 for SOFA tool. According to the given explanations as well as the cutoff points (with the highest percentage of accuracy) obtained for each tool, the ability of APACHE IV tool was higher to predict mortality in non-trauma patients, while SOFA tool was more appropriate for prediction of mortality in trauma patients.

In Table 3, estimation of the mortality risk ratio based on the Cox regression model has been presented by considering the length of stay in the hospital as the time variable by two tools of APACHE IV and SOFA, and has been analyzed based on three models.

**Table 3:** Estimation of the death risk ratio based on different models of scores of APACHE IV and SOFA tools in patients admitted to the ICU using the Cox regression

Variables			Trauma		Non-trauma		Total	
			HR (95%CI)	*P* value	HR (95%CI)	*P* value	HR^a^ (95%CI)	*P* value
APACHE IV	Model 1	Quantitative score	1.042 (1.029–1.055)	> 0.001	1.058 (1.043–1.073)	> 0.001	1.047 (1.038–1.057)	> 0.001
	Model 2 mean	> 49	Reference		Reference		Reference	
		≥ 49	4.821 (2.022–11.492)	> 0.001	10.54 (92.548–44.046)	> 0.001	6.214 (2.989–12.918)	> 0.001
	Model 3 one third	> 40	Reference		Reference		Reference	
		40–61	1.443 (0.360–5.784)	0.605	0.638 (0.090–4.540)	0.654	1.068 (0.349–3.269)	0.908
		≥ 61	9.100 (2.792–29.663)	> 0.001	9.500 (2.278–39.61)	> 0.002	9.281 (3.742–23.016)	> 0.001
SOFA	Model 1	Quantitative score	1.476 (1.343–1.623)	> 0.001	1.252 (1.157–1.355)	> 0.001	1.342 (1.266–1.422)	> 0.001
	Model 2 mean	> 3	Reference		Reference		Reference	
		≥ 3	9.055 (2.192–37.410)	> 0.002	3.071 (1.085–8.691)	0.035	5.151 (2.245–11.822)	> 0.001
	Model 3 one third	> 2	#		Reference		Reference	
		2–4	Reference		0.471 (0.112–1.982)	0.305	0.555 (0.150–2.054)	0.378
		> 4	15.022 (5.382–41.928)	> 0.001	3.689 (1.124–12.10)	0.031	6.191 (1.948–19.674)	> 0.002

^a^Hazard ratio

In the first model, on the basis of the quantitative score of the APACHE IV tool, with the increase in each 1 unite, the risk of death increased 4.2% in trauma patients and 5.8% in non-trauma ones, respectively.

In the second model, based on the quantitative score of the APACHE IV tool, by determining scores less than 49 as the reference, for scores equal to and more than 49, the risk of death increased by 4.8 times in traumatic patients and 10.5 times in non-traumatic ones, respectively.

In the third model, based on the quantitative score of the APACHE IV tool, scores were divided into three subsets of 0 to 40, 40 to 61, and 61 and more. Scores less than 40 were considered as the reference. Despite the insignificance of the results for scores 40 to 61, the risk of death in trauma and non-trauma people increased by 144% and 64%, respectively. However, it should be noted that by increasing the scores to 61 and more, the death probability increased 9.1 times in trauma patients and 9.5 times in non-trauma ones (*P* < 0.003).

In the first model, on the basis of the quantitative score of the SOFA tool, with the increase in each 1 unite, the probability of death increased by 4.7% in trauma people and 2.5% in non-trauma ones, respectively. In total, for every 1 unite increase in points of this tool, the risk of death increased 3.4%.

In the second model, based on the quantitative score of the SOFA tool, scores less than 3 were considered as the reference, and thus, for scores equal to and more than 3, the probability of death was 9.1 times in trauma patients and 3.1 times in non-trauma people, respectively. In general, at scores equal to and more than 3, the risk of death increased by 5.2 times.

In the third model, scores of 4 and less were considered as the reference and for scores more than 4, the risk of death increased 15.0 times in traumatic people and 3.7 times in non-traumatic ones, respectively.

#, in the model 3 of SOFA tool, in the first one-third, i.e., a score less than 2, there was no outcome of mortality, so the second one-third was introduced as a reference.

## Discussion

The study aimed to compare the power of SOFA and APACHE IV tools in predicting the mortality rate of patients in the ICU of Kowsar hospital in Sanandaj. According to the research, no study has been conducted in Iran with this title, purpose and the studied population on the two tools of SOFA and APACHE IV.

There was no significant difference between the outcomes of death and discharge between men and women (P=0.772). This result was in line with the ones of Sun et al. by which the study also did not show a significant relationship between death, discharge and sex (P=0.454).

The mean age of discharged and died traumatic patients were 40.5 and 64 years, respectively (*P* < 0.001).These rates were 61 and 71 in discharged and died non-traumatic patients, respectively (*P*=0.001). In general, there was a significant relationship between the mean age and the outcomes of discharge and mortality (*P* < 0.001). According to the study of Sun et al. [13], there was a significant relationship between age and the death outcome (*P*=0.001) so that the average age of deceased patients was 42.7 years and the average age of discharged ones was 35.1 years.

The mean length of stay in the hospital in discharged and deceased traumatic patients were 7.5 and 9.5 days, respectively (*P* = 0.180), while these rates were 6 and 9.5 days (*P* = 0.032) in discharged and died non-traumatic patients, respectively. In general, a significant association was found between the mean length of stay in the hospital and the outcomes of discharge and mortality (*P* = 0.012). In the study of Ghorbani et al. [14], the length of stay in the hospital in died patients was reported to be 14.4 days and in discharged ones, it was 10.3 days (*P* = 0.001).

The mean GCS was obtained 13 in discharged patients and 7 in deceased ones and in general, a significant relationship was found between the mean GCS and the outcomes of discharge and mortality (*P* < 0.001). According to the study of Ghorbani et al. [14], the mean GCS in discharged patients was 11.1 and it was 7.8 in deceased ones. A significant relationship was found between the mean GCS and the outcomes of discharge and mortality (*P* < 0.001).

The mean score of APACHE IV for discharged, and died patients was 44 and 81, respectively. In general, a significant relationship was observed between the mean score of APACHE IV tool and the outcomes of discharge and mortality in patients (*P* < 0.001). According to the study of Sun et al. [13], the mean score of APACHE IV in discharged patients was 11 and in deceased ones, it was 43. There was also a significant relationship between the APACHE IV score and the outcomes of discharge and mortality (*P* < 0.001). According to the study of Ghorbani et al. [14], the mean score of APACHE IV in discharged patients was 46.4 and this value was 81.9 in died ones (*P* = 0.001). By differentiating the results of trauma and non-trauma patients, the mean scores of APACHE IV tool in discharged and died trauma patients were observed 43 and 80.5, respectively (*P* < 0.001), while these scores in discharged and died non-trauma patients were obtained 46 and 85.5, respectively (*P* < 0.001).

The mean of mortality prediction percentage of APACHE IV in discharged and died traumatic patients were 6.7 and 44.2 (*P* < 0.001), respectively. These rates were 7.7 and 50 in discharged and died non-traumatic patients (*P* < 0.001), respectively. Overall, there was a significant relationship between the mean of mortality prediction percentage of APACHE IV and the outcomes of discharge and mortality in patients (*P* < 0.001). In the study of Ghorbani et al. [14], the observed mortality rate was 17.8% and the predicted mortality rate was 21% by APACHE IV (*P* = 0.036).

The mean score of SOFA for discharged patients was 3 and this rate was 8.5 for the deceased ones. A significant relationship was observed between the mean score of SOFA tool and the outcomes of discharge and mortality in patients (*P* < 0.001). According to the study of Mahjoubipour et al. [18], there was a significant relationship between the SOFA score of patients at the time of arrival and the outcome of death (*P* = 0.001) so that the higher the SOFA score at the time of arrival, the higher the involvement rate of 6 organs. Sun et al. [13] showed there was a significant relationship between the SOFA score and the outcome of death (*P* < 0.001). The mean score of SOFA in deceased patients was 8 and it was 2 in discharged ones. Saleh et al. [6] showed there was a significant relationship between the SOFA score and the outcome of death (*P* = 0.001), while the means of the SOFA score were reported 9 in died patients and 4 in discharged ones. By differentiation results in trauma and non-trauma patients, the mean score of the SOFA tool was observed 3 and 9 (*P* < 0.001) in discharged and deceased trauma patients as well as 3 and 7 in discharged and died non-trauma patients, respectively (*P* < 0.001).

The mean of mortality prediction percentage of SOFA tool was 5 and 20 (*P* < 0.001) in discharged and died trauma and non-trauma patients, respectively (*P* < 0.001). A significant relationship was observed between the mean of mortality prediction percentage of SOFA tool and the outcomes of discharge and mortality (*P* < 0.001). In the study of Saleh et al. [6], the mortality prediction percentage of SOFA tool was 20–10% in deceased patients and < 10% in discharged ones.

In the first model, by evaluating the hazard ratio quantitatively considered as tool scores, it was found that in both APACHE IV and SOFA, with increasing the death prediction score, the death hazard also increased significantly. It was important that, like the previous findings, the hazard ratio in APACHE IV and SOFA was higher for non-traumatic and traumatic patients, respectively. In the present study, the hazard ratios for quantitative scores of both APACHE IV and SOFA in the ICU patients were 1.05 and 1.34, respectively. In the only similar study conducted by Sun et al., the hazard ratios for quantitative scores of both APACHE IV and SOFA in patients with acute myocarditis were, respectively, 1.09 and 1.53 [13] slightly higher than the findings of this study in both tools.

In the second model, the mean score of the 2 tools was considered as the cutoff point and the scores less than the mean were considered as references. Like the previous findings, the hazard ratios in APACHE IV and SOFA were higher for non-trauma (HR = 10.55) and trauma patients (HR = 9.06), respectively. The hazard ratios for all patients in the cutoff point model based on the mean were 6.21 and 5.15 for APACHE IV and SOFA tools, respectively, while in the study of Sun et al., they were 7.95 and 6.80 [13] slightly higher than the findings of this study for both tools. In the third model, the scores of the two tools were divided into three parts and the smaller part was considered as the reference. Like the previous findings, the hazard ratios of the upper one-third were higher in both APACHE IV and SOFA, for non-trauma (HR = 9.50) and trauma patients (HR = 15.02), respectively. In the third model, the hazard ratios of the upper one-third were 9.28 and 6.19 in both APACHE IV and SOFA for all patients, respectively. In the study of Sun et al., they were 33.86 and 12.16 for both APACHE IV and SOFA tools, respectively [13], which were higher than the findings of this study in the both tools.

The findings of the APACHE IV and SOFA scoring systems in traumatic and non-traumatic ICU patients showed a higher ability of mortality prediction for APACHE IV. However, in the separated comparison of this rate in trauma and non-trauma patients, we concluded that the ability to predict the mortality of trauma patients in SOFA tool was higher than that in APACHE IV and also the ability to predict mortality of non-trauma patients in APACHE IV was higher than that in SOFA tool. According to the above, APACHE IV was more suitable for non-traumatic patients and SOFA tool for traumatic ones.

The area under the rock curve was 0.902 for APACHE IV tool at the cutoff point of 74 with the highest percentage of accuracy (90.35%), sensitivity (69.77%) and specificity (95.91%). This value was obtained 0.895 for SOFA tool at the cutoff point of 7 with the highest percentage of accuracy (91.34%), sensitivity (75.58%) and specificity (95.60%). However, the results of alignment were also seen in other studies. In the study of Sun et al. [13], the area under the rock curve was reported 0.934 for APACHE IV tool at the cutoff point with sensitivity of 83% and 0.920 for SOFA tool at the cutoff point with sensitivity of 78%. In the study of Ibrahimi et al. [9], the area under the rock curve was 0.808 for APACHE IV tool and 0.897 for sofa tool. Other studies showed APACHE IV criteria had sensitivity of 69.57% and specificity of 91.04% in predicting mortality of the ICU patients [19]. Furthermore, in another study, results lower than those of the present study were seen so that the SOFA scoring system had sensitivity of 37% and specificity of 79% in the prediction of mortality of hospitalized patients suspected of sepsis [20]. In this study, by differentiating traumatic and non-traumatic patients, the areas under the rock curve were found 0.893 in traumatic patients and 0.918 in non-traumatic ones for APACHE IV, while they were 0.940 in trauma patients and 0.845 in non-trauma ones for SOFA.

Among the weaknesses of the study, we can point out the lack of evidence and information in the documents of some patients, which has caused the exclusion of these patients from the study process. One of the strong points of this study is the separation of patients into two trauma and non-trauma categories, which has led to the extraction of new findings in this field.

## Conclusions

Based on the results of this study, very small difference was found between APACHE IV and SOFA tools in predicting mortality of the ICU patients. However, if trauma and non-trauma patients were separately examined, APACHE IV performance was better in predicting mortality of non-traumatic patients and SOFA performance in predicting mortality of traumatic ones. Therefore, these two tools are applicable for different groups of patients to achieve better quality care and treatment.

## Data Availability

The used data are always available and provided when needed.

## Abbreviations

AIDS: Acquired immunodeficiency syndrome
APACHE: Acute physiology and chronic health evaluation
AUC: Area under the curve
CRF: Chronic renal failure
FiO2: Fraction of inspired oxygen
HR: Hazard ratio
ICU: Intensive care unit
IQR: Inter-quartile range
GCS: Glasgow coma scale
MPM: Mortality probability models
PaO2: Partial pressure of oxygen
PCO2: Partial pressure of carbon dioxide
PH: Potential of hydrogen
ROC Curves: Receiver operating characteristic curve
SAPS: Simplified acute physiology score
SOFA: Sequential organ failure assessment
SPSS: Statistical package for the social sciences

## Footnotes

1 Glasgow Coma Scale.

2 Simplified Acute Physiology Score.

3 Acute Physiology And Chronic Health Evaluation.

4 Mortality Probability Models.

5 Sequential Organ Failure Assessment.

6 Acute Physiology And Chronic Health Evaluation (II).

7 Acute Physiology And Chronic Health Evaluation (IV).

8 Chronic renal failure.

9 Acquired Immunodeficiency Syndrome.

10 area under the curve.
